# Influence of cutaneous and muscular circulation on spatially resolved versus standard Beer–Lambert near‐infrared spectroscopy

**DOI:** 10.1002/phy2.179

**Published:** 2013-12-05

**Authors:** Alessandro Messere, Silvestro Roatta

**Affiliations:** 1 Department Neuroscience, University of Torino, Torino, Italy

**Keywords:** Muscle blood flow, muscle blood volume, muscle oxygenation, near‐infrared spectroscopy, skin blood flow

## Abstract

The potential interference of cutaneous circulation on muscle blood volume and oxygenation monitoring by near‐infrared spectroscopy (NIRS) remains an important limitation of this technique. Spatially resolved spectroscopy (SRS) was reported to minimize the contribution of superficial tissue layers in cerebral monitoring but this characteristic has never been documented in muscle tissue monitoring. This study aims to compare SRS with the standard Beer–Lambert (BL) technique in detecting blood volume changes selectively induced in muscle and skin. In 16 healthy subjects, the biceps brachii was investigated during isometric elbow flexion at 70% of the maximum voluntary contractions lasting 10 sec, performed before and after exposure of the upper arm to warm air flow. From probes applied over the muscle belly the following variables were recorded: total hemoglobin index (THI, SRS‐based), total hemoglobin concentration (tHb, BL‐based), tissue oxygenation index (TOI, SRS‐based), and skin blood flow (SBF), using laser Doppler flowmetry. Blood volume indices exhibited similar changes during muscle contraction but only tHb significantly increased during warming (+5.2 ± 0.7 *μ*mol/L·cm, an effect comparable to the increase occurring in postcontraction hyperemia), accompanying a 10‐fold increase in SBF. Contraction‐induced changes in tHb and THI were not substantially affected by warming, although the tHb tracing was shifted upward by (5.2 ± 3.5 *μ*mol/L·cm, *P* < 0.01). TOI was not affected by cutaneous warming. In conclusion, SRS appears to effectively reject interference by SBF in both muscle blood volume and oxygenation monitoring. Instead, BL‐based parameters should be interpreted with caution, whenever changes in cutaneous perfusion cannot be excluded.

## Abstract (precis)

The influence of cutaneous circulation on muscle NIRS monitoring has been seldom investigated and is often overlooked. This study shows that cutaneous dilatation induced by superficial warming produces consistent changes in standard Beer–Lambert (BL) parameters while leaving spatially resolved parameters unaffected. Thus, Beer–Lambert parameters should be interpreted with caution whenever changes in cutaneous perfusion are expected to occur.

## Introduction

Near‐infrared spectroscopy (NIRS) can provide potentially useful information about changes in blood volume and tissue oxygenation (Perrey 2008; Ferrari et al. 2011; Hamaoka et al. 2011; Ghosh et al. 2012). However, its employment and usefulness are still limited due to several reasons among which is the poorly defined influence of superficial tissue layers (skin) on NIRS variables, when aiming to assess changes occurring in deep tissues, such as brain and muscle. This is a relevant issue in cerebral monitoring as control of skin blood flow is considerably different from cerebral blood flow and can introduce important alterations in the measurement (Lam et al. 1997; Smielewski et al. 1997; Canova et al. 2011). The issue is relevant also in muscle monitoring as exercise is often accompanied by an increase in local and whole body temperature that may be associated with relevant changes in cutaneous blood flow (Roberts and Wenger 1979; Kenney and Johnson 1992), although its potentially confounding effects have been often overlooked.

Increasing the distance between emitting and detecting optodes is known to increase the depth of penetration of infrared light (Cui et al. 1991; McCully and Hamaoka 2000); however, this expedient minimizes but does not exclude superficial tissues from the sampling volume. Among the several computational improvements in NIRS signal processing, as implemented in the different commercial devices, only few of them address the problem of minimizing the influence of cutaneous perfusion. Spatially resolved spectroscopy (SRS) was developed with the aim of providing quantitative assessment of tissue of oxygenation (Matcher et al. 1995; Suzuki et al. 1999) but was subsequently observed to minimize the contribution of superficial versus deep tissue layers (Kirkpatrick et al. 1998; Al‐Rawi et al. 2001). The technique is based on collecting the light signals at 2–3 different points close to each other but located at slightly different distance from the light emitter. The light paths leading from the emitter to the different detectors share a common part, the one related to superficial tissues, while the path length in the deep tissues is longer for the detectors at larger distance. Thus, by analyzing the differential signal collected by the detectors, SRS variables are intrinsically more sensitive to the changes occurring in deep tissue layers, as compared with variables computed according with the standard modified Beer–Lambert law (BL).

In this respect, the better performance of SRS as compared to BL methodology has been recently documented in healthy subjects undergoing cerebral NIRS monitoring during standard neurovegetative tests (Canova et al. 2011). This study showed that (1) cutaneous circulation differently affects BL and SRS blood volume measurements, to the extent that changes of opposite sign were detected for the same test in 31% to 46% of the subjects, depending on the test employed, (2) BL measurements better correlate with indicators of extracranial blood volume, as compared to SRS measurements (Canova et al. 2011).

Only few studies investigated the influence of skin circulation on NIRS measurements in skeletal muscles, globally providing non univocal indications (Mancini et al. 1994; Buono et al. 2005; Davis et al. 2006; Tew et al. 2010) and, surprisingly, SRS and BL methodologies have never been compared in their ability to focus on muscle rather than skin tissue.

We also hypothesized that in skeletal muscle SRS monitoring would be less sensitive to cutaneous circulation than BL and that the BL‐SRS comparison could help in understanding the contribution of cutaneous circulation to the overall NIRS monitoring.

To test this hypothesis SRS and BL variables were monitored during maneuvers exclusively affecting muscle and skin perfusion, that is, muscle contraction and local superficial warming, respectively, in healthy subjects. As in the study mentioned above (Canova et al. 2011), the comparison was focused on changes exhibited by total blood volume indicators THI (SRS) and tHb (BL), which are simultaneously provided by a NIRS device, implementing both SRS and BL methodologies. Specific indices were devised to quantify the contribution of cutaneous versus muscle circulation to the measurement.

## Methods

### Subjects

Sixteen healthy subjects (11 men and 5 women) were enrolled in the study. The mean age, height, and weight were 27.3 ± 4.4 years, 174.6 ± 9 cm, and 67.1 ± 12.8 kg, respectively, and adipose tissue thickness over the right biceps muscle was 2.9 ± 0.4 mm. The study was approved by the Local Ethical Committee and all subjects gave their informed consent.

### Experimental setup

NIRS monitoring was performed using a continuous wave NIRS system (NIRO‐200NX, Hamamatsu Photonics, Hamamatsu City, Japan), which simultaneously implement BL and SRS methods (Delpy et al. 1988; Suzuki et al. 1999; Wolf et al. 2007).

BL parameters provide a measure of concentration changes in oxyhemoglobin (O_2_Hb), deoxyhemoglobin (HHb), and total hemoglobin (tHb = O_2_Hb + HHb) with respect to an arbitrary initial value, and are expressed in *μ*mol/L·cm. These measures could be converted to *μ*mol/L through multiplication by the interoptode distance (4 cm in this study) and the *path‐length factor*. As for SRS, two parameters are provided, one gives information about tissue oxygenation (TOI, total oxygenation index), it is expressed in % and represents the percentage ratio of oxygenated hemoglobin to total hemoglobin. The other parameter is again a measure of total hemoglobin contents in the tissue (THI, total hemoglobin index) and is expressed in arbitrary units. Different from BL parameters, changes in SRS parameters may be expressed in relative terms (e.g., as % of baseline or resting level). Note that NIRS cannot discriminate between hemoglobin and cytoplasmatic myoglobin, therefore, all measurements actually refer to [hemoglobin + myoglobin] in the sample volume (Spires et al. 2011). However, as the myoglobin concentration is not supposed to change, all concentration changes can be attributed to blood volume changes.

The NIRS probe (interoptode distance = 4 cm) was attached to the skin at the medial aspect of the biceps brachii muscle with double‐sided adhesive tape.

Skin blood flow was measured using a laser Doppler flowmeter (Periflux PF 2, Perimed KB, Stockholm, Sweden), whose cylindrical probe PF 302 (OD 2.2 mm) was positioned over the belly of the right biceps brachii, 2 cm proximal to the NIRS probe, by means of a plastic holder stuck to the skin.

Local warming of the skin was obtained by exposing the frontal surface of the upper arm to warm air (*t* = 43°C; KX2200K, Black & Decker, Towson, MD) (Sessler and Moayeri 1990; Blaak et al. 1992; Giesbrecht et al. 1994; Ohtsuka et al. 2002). Skin temperature was measured with a digital thermometer (Omega 450‐ATH, Stamford, CT) positioned in‐between NIRS and flowmeter probes.

### Experimental protocol

All measurements were performed in a quiet room with a constant ambient temperature of about 20°C. The subject was seated upright on an adjustable chair with the back supported by a back rest. The right forearm was positioned on a horizontal support, with an elbow angle of about 120° and the hand, oriented with the palm up, was resting below the handle of a force transducer (TF 02, CCt Transducers, Torino, Italy).

After positioning all probes, the maximum voluntary contraction (MVC) was determined as the peak force recorded during three 3‐sec lasting maximal isometric elbow flexions, separated by 2‐min resting intervals.

After 10 min of rest the subject was asked to perform an isometric elbow flexion at 70% of MVC, lasting 10 sec. To this purpose a visual feedback of the force intensity was provided to the subject who was asked to match as steadily as possible the force level indicated by a horizontal cursor.

The warming phase was started 2–3 min after the end of the first contraction, producing a gradual increase in skin blood flow up to a plateau level. Thirty seconds after reaching the plateau level the subject was asked to perform a second elbow flexion (70% MVC, 10 sec). The air flow was stopped 1 min later, lasting in total 6–9 min, depending on the time required for the SBF to reach the plateau level. Skin temperature was measured twice: 10 sec before the beginning and 20 sec after the end of the warming phase.

In order to assess the possible thermal drift of the NIRS sensors, the warming procedure was repeated after positioning the NIRS probe over a piece of fresh meat (a 7 cm thick pork tenderloin). Duration of the exposure to warm air lasted 7 min and was repeated four times in 4 h.

### Data acquisition and analysis

The NIRS device provides an analog output for all variables, which was digitally acquired along with skin blood flow and force signals (CED Micro 1041, Cambridge Electronic Design, Cambridge, UK; sampling frequency = 50 Hz) and stored on the computer for later analysis with Spike2 software (version 6.10, Cambridge Electronic Design, UK).

**Figure 1.**
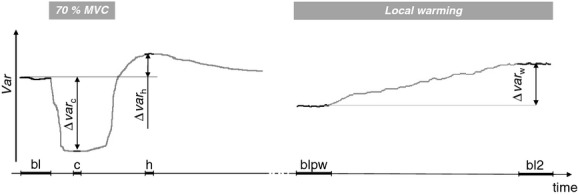
Illustration of the time intervals and of the notation used for the measurements (see text). The drawing represents the hypothetical response of a generic variable *Var* to contraction (left) and local warming (right).

NIRS variables were measured as time averages over selected intervals (Fig. 1): bl1 (Baseline: 20‐sec interval located just before the first contraction), c1 (2‐sec interval centered on the minimum value reached during the first contraction), h1 (2‐sec interval, centered on the peak of the postcontraction hyperemia), blpw (prewarming baseline: 20‐sec interval located just before the warming phase) and bl2, c2 and h2, defined as bl1, c1 and h1, respectively, for the second contraction.

On the basis of these readings, the effects produced on the generic variable (*var*) were computed as follows (see also Fig. 1): warming (Δ*var*_w_ = *var*_bl2_ − *var*_blpw_), contraction 1 (Δ*var*_c1_ = *var*_c1_ − *var*_bl1_), hyperemia 1(Δ*var*_h1_ =*var*_h1_ − *var*_bl1_), contraction 2 (Δ*var*_c2_ = *var*_c2_ − *var*_bl2_) and hyperemia 2 (Δ*var*_c2_ = *var*_c2_ − *var*_bl2_). All these effects are computed as absolute changes and are thus expressed in *μ*mol/L·cm for tHb, in% of control (bl1) for THI, and in% of tissue oxygen saturation for TOI.

Finally, in order to provide a quantification of the dependence of blood volume measurements on skin and muscle blood perfusion and in order to make the performance of tHb and THI comparable, two *skin interference indices* (SII) were defined as the ratio of the response to warming and the response to contraction expressed in percentage. For the latter term either the volume decrease recorded during the contraction or the volume increase recorded during the subsequent hyperaemia could be considered, resulting, respectively, in: (1) SIIc_*var*_ = Δ*var*_w_/(−Δ*var*_c1_) × 100, which represents how large is the blood volume increase obtained by local warming, as compared to the decrease observed during the contraction and (2) SIIh_*var*_ = Δ*var*_w_/Δ*var*_h1_ × 100 along the same line, which represents how large is the blood volume increase obtained by local warming, as compared to the increase observed during the postcontraction hyperaemia. In order to assess the effect of the warming maneuver on the NIRS probe only Δ*tHb*_w_ and Δ*THI*_w_ were evaluated.

### Statistics

Statistical analysis was performed using the SPSS Statistics software version 20 (IBM, Armonk, NY). Significant changes with respect to baseline were assessed through nonparametric statistics for repeated measures (Friedman ANOVA and Wilcoxon post hoc test) as some of the variables failed to exhibit a normal distribution.

The effect of warming on skin blood flow, skin temperature, Δ*var*_c_, and Δ*var*_h_ were assessed by the Wilcoxon test for repeated measures.

In addition, the comparison between BL and the SRS parameters in terms of SII indices was also performed by the Wilcoxon test. All data are presented as mean ± SD, while in figures error bars indicate standard error.

## Results

**Figure 2.**
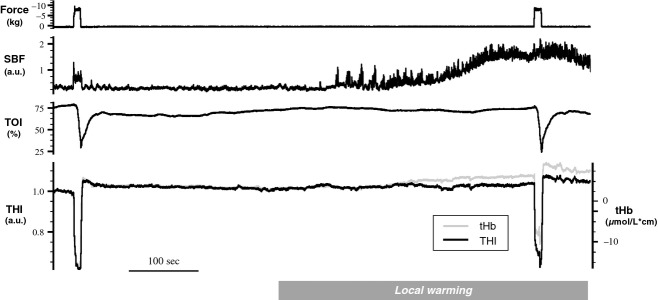
Original tracings from a representative subject. From top to bottom: force, skin blood flow (SBF), tissue oxygenation index (TOI), and blood volume indices (THI in black and tHb in gray). The subject performs two contractions at 70% of MVC, as indicated by the force signal, before and after a stable level of cutaneous hyperemia is reached, following exposure to warm air flow (local warming indicated at the bottom). Amplifications of THI and tHb was adjusted in order to have overlapping responses to the first muscle contraction. Note how the tHb signal is affected by changes in SBF, as compared to THI.

**Figure 3.**
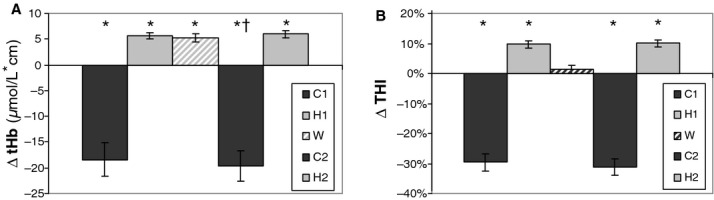
Average blood volume changes detected by tHb (A) and THI (B) recorded during the first contraction (C1) and postcontraction hyperaemia (H1), after stabilization of warming‐induced cutaneous hyperemia (W) and during the second contraction (C2) and postcontraction hyperaemia (H2). Error bars indicate the standard error (*n* = 16). (*) significantly different from 0 (*P* < 0.01); (†) contraction 2 versus contraction1, *P* < 0.05.

The MVC was on average 14.9 ± 6.0 kg. Figure 2 shows the response of a representative subject to the full experimental protocol. It can be observed that during the first contraction, both blood volume indices exhibit overlapping variations consisting of a marked reduction followed by a small increase. The extent of reduction was on average 18.4 ± 12.3 *μ*mol/L·cm for tHb and 29.5 ± 11.3% for THI, while the maximum increase displayed during the postcontraction hyperaemia was on average 5.6 ± 2.4 *μ*mol/L·cm for tHb and 9.7 ± 4.8% for THI (Fig. 3). Skin blood flow only apparently increased during contractions (Fig. 2), in fact, both the sharp changes at onset and release of the contraction, and the immediate return to precontraction level indicate that the effect has to be interpreted as a movement artifact.

The total duration of the warm air exposure was 7.2 ± 1.7 min. During warming, skin blood flow gradually increased up to a plateau level, reaching on average a 1169 ± 367% of control (*P* < 0.01), while skin temperature increased from 32.6 ± 1.0 to 36.7 ± 0.5°C (*P* < 0.01).

With respect to the prewarming level, tHb exhibited on average a consistent increase (ΔtHb_w_ = 5.2 ± 0.7 *μ*mol/L·cm, *P* < 0.01). Such increase in blood volume results from significant increases of both HHb (1.06 ± 1.0*μ*mol/L·cm, *P* < 0.01) and O_2_Hb (4.16 ± 3.3 *μ*mol/L·cm, *P* < 0.01). Instead, THI decreased in 5 of 16 subjects resulting on average in a nonsignificant change (ΔTHI_w_ = 1.4 ± 5.3%).

It can be observed that the tHb response to the second contraction appears to be shifted upward with respect to the previous contraction (Fig. 2). On average, the minimum level reached during the second contraction was significantly higher than that in the first contraction: tHb_c2_ − tHb_c1_ = 5.2 ± 3.5 *μ*mol/L·cm (*P* < 0.01). This difference closely matches the increase exhibited during warming, ΔtHb_w_. No significant change was exhibited by THI (THI_c2_ − THI_c1_ = 2.0 ± 6.2, *P* > 0.05).

Blood volume changes observed during the second contraction and postcontraction hyperaemia were 19.6 ±11.5 and 6.0 ± 2.6 *μ*mol/L·cm, respectively, for tHb and 31.1 ± 10.7% and 10.1 ± 4.9%, respectively, for THI. The differences between the second and the first contraction were in general rather small (<7%) and reached statistical significance only for tHb_c_ (*P* < 0.05).

**Figure 4.**
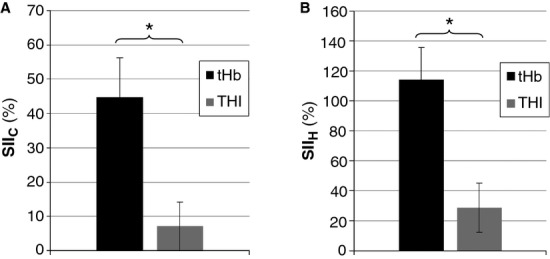
Comparison of skin interference indices (SII) for tHb and THI. SIIc is obtained by the ratio of the blood volume increase exhibited during cutaneous hyperemia and the decrease during contraction (A), SIIh is obtained by the ratio of blood volume increase exhibited during cutaneous hyperemia and the increase during postcontraction hyperemia (B), both being expressed in percentage. See text in the Methods section for further explanations. Error bars indicate the standard error (*n* = 16). (*) *P* < 0.05.

The different dependence on skin perfusion for tHb and THI, as quantified by the skin interference indices (i.e., SII_c_ and SII_h_), is displayed in Figure 4. It can be observed that tHb exhibits significantly higher values than THI for both SIIc: 44.8 ± 44.6% versus 7.1 ± 29.5% (*P* < 0.01) and SII_h_ 114.2 ± 83.8% versus 28.6 ± 63.8% (*P* < 0.01). Note that an SII_h_ value in the order of 100% means that tHb exhibits comparable changes in response to local warming and muscle contraction (see also Fig. 3A).

Finally, when the warming procedure was performed on the dummy subject (the pork tenderloin), changes detected on NIRS parameters were: ΔtHb_w_ = −0.3 ±0.2 *μ*mol/L·cm (*P* < 0.05), ΔTHI_w_ = 0.1 ± 0.2% (*P* < 0.05) and ΔTOI_w_ = 0.4 ± 0.2% (*P* < 0.05). Note that, although reaching statistical significance, these effects are an order of magnitude smaller than the effects observed on human subjects. This excludes a major role of temperature drift in the observed results.

The TOI exhibited a gradual decrease during the first contraction from a resting level of 73.8 ± 6.2% to a minimum value of 34.4 ± 14.8% (*P* < 0.01). The warming procedure did neither affect the resting oxygenation level (ΔTOI_w_ = 1.4 ± 4.3%) nor the response to the contraction (second contraction: baseline: 75.2 ± 5.9%; contraction 33.5 ± 18.6%).

## Discussion

In this study we observed that local warming of the forearm results in detectable alteration of blood volume NIRS parameters, not attributable to thermal drift of the probes. This effect is consistently observed in the BL parameters tHb, O_2_Hb, and HHb. In tHb, the extent of the increase during warming was comparable with the increase observed in the hyperemia produced by a 10‐sec contraction at 70% of MVC. Conversely nonsignificant changes were exhibited by the SRS parameters THI and TOI: this indicates a larger influence of cutaneous circulation on BL than SRS parameters, which confirms our initial hypothesis. This dependence, quantified by SII indices, resulted in four to six times greater values for tHb than for THI.

### SRS versus BL in response to muscle contraction and local warming

The experimental protocol used in this study was aimed at inducing circulatory changes selectively in muscle and skin. Both parameters showed the same expected pattern of response to muscle contraction. A marked and prompt decrease in blood volume was exhibited during the contraction, corresponding to the depletion of blood from intramuscular blood vessels that are squeezed by the increase in intramuscular pressure (Laughlin 1987; Ferrari et al. 2011), although movement artifacts and other factors may also contribute. Such increase in intramuscular pressure also stops blood flow to the muscle resulting in a transient ischemia, which, in combination with the increased metabolism gives rise to the postcontraction hyperemia (Felici et al. 2009; Ferrari et al. 2011). Due to the short duration of the contraction, this hyperemic phase is associated with a mild increase in blood volume. However, the reason for choosing a short contraction duration was to reduce muscle fatigue and allow for fast recovery, thus limiting the development of systemic effects on cardiovascular variables (see later).

The results of this study indicate that both NIRS methodologies effectively detected changes in muscle blood volume, providing qualitatively similar responses.

On the other hand, direct warming of the skin is known to cause selective cutaneous vasodilatation (Barcroft and Edholm 1943; Johnson and Rowell 1975; Hodges et al. 2009; Johnson and Kellogg 2010). The response of skin blood flow to local warming produces a typical response pattern, that is, skin blood flow markedly increases when local temperature exceeds a certain threshold (about 37°C) and, if the warming stimulus is maintained, it stabilizes at a plateau level (Barcroft and Edholm 1943; Kellogg et al. 1995; Minson et al. 2002; Hodges et al. 2009; Johnson and Kellogg 2010). Skin blood flow recordings in this study confirm this pattern (Fig. 2). According to the literature, changes of SBF or cutaneous vascular conductance in the order of 300–1100% are frequently reported even during mild and “non extreme” exercise such as cycling (Fritzsche and Coyle 2000; Yamazaki and Sone 2003, 2006; Journeay et al. 2005; Wang 2005) and arm cranking (Theisen et al. 2001), as well as during body heating (Johnson 1986; Kellogg et al. 1999). The significant blood volume changes detected by tHb and not by THI at the end of the warming phase reveal that the former parameter is affected by cutaneous dilatation. The observation that most of the warming‐induced increase in tHb results from the increase in O_2_Hb fits well with an increase in skin blood flow and blood volume that occur at unchanged metabolism. Considering that the increase in tHb could start long before SBF reached the plateau level (Fig. 2), it is suggested that skin perfusion may affect BL measurements in a wide range of conditions in which thermoregulatory adjustments occur. It is worth underlying that both measurements are simultaneously obtained from the same sample volume, same interoptode distance, same laser wavelengths, thus the different sensitivity to cutaneous circulation must be attributed to the different processing methodology.

The potential influence of skin circulation on muscle NIRS measurements has been investigated in previous studies reporting conflicting results (Mancini et al. 1994; Buono et al. 2005; Davis et al. 2006). Although the investigation by Mancini et al. (1994) suggested little dependence on skin blood flow, the studies by Buono et al. (2005) and Davis et al. (2006) reported significant alterations of NIRS parameters by changes in skin circulation. However, these latter observations were likely affected by the use of a short interoptode distance (25 and 20 mm, respectively) which reduces the depth of the sample volume, as compared to larger distances (i.e., 40–50 mm). More recently, Tew et al. (2010) re‐investigated the issue by simultaneously looking at both blood volume (tHb, based on BL methodology) and tissue oxygenation (SmO_2_ = TOI, based on SRS) parameters during rest and dynamic knee extension trials performed at different extent of thigh heating (i.e., no heating, 37, and 42°C skin temperature). They observed that local warming affected both parameters at rest while only tHb exhibited an increase also during exercise. On this basis they concluded that NIRS‐derived measures of tissue oxygenation and blood volume are differentially affected by skin blood flow, leaving the question open as to whether the different responses were to be attributed to the different nature of the measured variables or to the different underlying computational methodologies (BL vs. SRS) (Tew et al. 2010). The present results, which partly confirm and integrate the above observations, support the latter possibility.

First of all, we also observed significant increase in tHb during warming, and increased tHb levels in response to the muscle contraction when it is performed after warming. Note that we observed additive tHb responses to local warming and muscle contraction thus confirming the hypothesis that tHb integrates blood volume changes taking place in both skin and muscle tissues. In their study Tew et al. (2010), found a less‐than additive combination of the two contributions, however, in their case the large muscle mass recruited (knee vs. wrist extensors) and the longer exercise duration (3 min vs. 10 sec) could have caused an increase in sympathetic activity and limited the cutaneous dilatation during the exercise (Johnson 1992). This can possibly explain their observation of smaller increase in (skin + muscle) blood volume during exercise in warm versus neutral conditions (Tew et al. 2010).

Second, different from this study, Tew et al. (2010) report a small increase in TOI during local warming, while we observe no significant changes in any of the SRS parameters. This might again be attributed to differences in the experimental conditions, in particular to the fact that also muscle blood vessels dilate in response to local increase in temperature. In fact, it has been recently observed in a PET study that an increase in muscle temperature from 33.4 to 37.4°C and muscle blood flow by 45%, after local warming (Heinonen et al. 2011). It is possible that exposing the limb to a 30‐min warming by means of an electrically powered heating pad (37–42°C) wrapped around the thigh (Tew et al. 2010) partly heated the most superficial muscle layers (those mostly concerned by the NIRS measurement) thus increasing muscle blood flow and TOI. Considering that heating by air flow is characterized by a lower rate of increase in core temperature than heating by direct contact (Wadhwa et al. 2007), it is likely that the heating procedure adopted in this study (exposure to warm air flow for 6.0 ± 1.7 min) did not substantially affect muscle blood flow. If this reasoning holds true, in both the present and Tew et al.'s studies SRS parameters exclusively indicated the actual changes occurring in skeletal muscles.

### Skin interference indices

The SII indices (SII_h_ and SII_c_) were here introduced with the double aim of (1) quantifying the dependence of NIRS parameters on cutaneous versus muscular circulation and (2) comparing the performance of the two blood volume parameters, tHb and THI, that cannot be directly compared as the former provides Hb concentration changes with respect to an arbitrary initial level, while the latter indicates relative changes. In particular, these indices relate the *skin‐dependent* blood volume change detected during warming to the *muscle‐dependent* change detected during (SII_c_) or immediately after (SII_h_) the contraction: the higher the index value, the higher the dependence on cutaneous circulation. As mentioned above, during the contraction muscle blood volume promptly and markedly decreases due to the increase in intramuscular pressure that squeezes intramuscular vessels, although red blood cells may also partly remain entrapped in capillaries (Gray et al. 1967). However, even in isometric conditions, the muscle shortens thus potentially altering the geometry of the NIRS optodes that are stuck over the muscle belly. In particular, this generally results in increased convexity of the cutaneous surface potentially leading to reduced attenuation of the infrared light beam and to a decrease in the estimated blood volume. Due to the occurrence of this possible movement artifact of unknown magnitude, besides SIIc we also considered the blood volume change occurring in the postcontraction hyperemia: in this condition the muscle is relaxed and the optode geometry is the same as in the resting precontraction condition. The drawback in this case being that a smaller blood volume change was generally observed that increased the variability in the SII_h_ index.

Besides these limitations, both SII_h_ and SII_c_ were significantly higher for tHb than for THI, thus further supporting the hypothesis of larger dependence of BL than SRS parameters on cutaneous circulation.

### The response to muscle contraction at different levels of cutaneous perfusion

As discussed above BL parameters were shown to be affected by changes in cutaneous perfusion but could a constant skin perfusion also affect the NIRS assessment of changes in muscle blood volume and oxygenation related to muscle contraction? To answer this question the changes detected by NIRS variables in response to the 10‐sec, 70% MVC contraction were compared before and after warming, that is, in stable conditions of low and high SBF.

The results showed that all differences between the two contractions were below 7%, meaning that even high levels of cutaneous perfusion provide only a minor interference in the assessment of blood volume changes taking place in the muscle. These small differences reached significance only for tHb and not for THI, further underlying the better performance of SRS over BL in rejecting interference from cutaneous circulation.

### Practical implications

Regulations of muscular and cutaneous circulation respond to different needs, primarily the metabolic needs of muscle activity and the thermoregulation of the body, respectively. These two functions are, however, interrelated so the problem of cutaneous interference in NIRS muscle monitoring cannot be easily dismissed.

Prolonged muscular work is associated with a high generation of metabolic heat load which increases body temperature. When internal temperature rises and reaches a threshold level, thermoregulatory mechanisms are activated in order to prevent overheating (Johnson 1986, 1992; Kellogg et al. 1991). These include extensive cutaneous vasodilatation and increase in SBF which, by raising the temperature of the body surface, improves heat dissipation (Johnson and Park 1981; Kellogg et al. 1991). Thermoregulatory adaptation of skin perfusion is thus expected to occur whenever a high‐power exercise is performed for a sufficient amount of time. In this case, NIRS monitoring of blood volume should better rely on SRS rather than standard BL methodology in order to avoid interference from changes in SBF. When monitoring exercises of low power/short duration changes in SBF are less likely to occur, BL parameters would instead detect contraction‐related changes independently of current level of cutaneous perfusion. As for muscle oxygenation, it is often assessed by monitoring changes in HHb (Grassi et al. 2003). The present results show that HHb is little affected by superficial warming confirming that this approach is rather refractory to cutaneous contamination (Grassi et al. 2003), although consideration of TOI would be preferable in this respect.

Other NIRS methodologies (e.g., time‐resolved and phase‐modulated) have been implemented in commercially available devices introducing the possibility of assessing changes in the scattering coefficient *μ*s', which is otherwise assumed to be constant (as in this study). The coefficient *μ*s' is related to the light scattering properties of the tissue and has been shown to exhibit wavelength‐dependent changes during strenuous exercise (Ferreira et al. 2007). The authors also showed that neglecting changes in *μ*s' may lead to overestimate changes in blood volume (Ferreira et al. 2007). We consider unlikely that relevant changes in *μ*s' have occurred in the present experimental protocol, which included only short lasting contractions and superficial warming, although the possibility cannot be excluded.

On the other hand the sensitivity of time‐ and frequency‐resolved spectroscopy to cutaneous circulation also remains to be ascertained. Comparative studies between different devices and methodologies should be carried out to elucidate these issues.

## Conclusions

The results of this study allow to draw the following conclusions: (1) changes in cutaneous circulation can markedly affect NIRS variables based on the BL law, even if large (4‐cm) interoptode distance is used; (2) the magnitude of the interference produced by skin vasodilation is comparable to blood volume changes produced by the hyperemia that follows an isometric contraction of 10‐sec duration, at 70% MVC; (3) SRS‐based variables appear to be much less sensitive than BL variables to changes in skin circulation, in accordance with the hypothesis.

Based on the above consideration, the adoption of SRS variables rather than BL variables is recommended whenever changes in skin perfusion, such as the ones originated by thermoregulation or stress, are expected to occur during NIRS monitoring.

## Acknowledgments

We are grateful to the Laboratory of Engineering of Neuromuscular System and Motor Rehabilitation (LISiN, Politecnico di Torino) for lending us the NIRS device.

## Disclosures

None declared.
